# Hematological toxicities in PARP inhibitors: A real‐world study using FDA adverse event reporting system (FAERS) database

**DOI:** 10.1002/cam4.5062

**Published:** 2022-07-24

**Authors:** Yamin Shu, Yufeng Ding, Xucheng He, Yanxin Liu, Pan Wu, Qilin Zhang

**Affiliations:** ^1^ Department of Pharmacy, Tongji Hospital, Tongji Medical College Huazhong University of Science and Technology Wuhan China; ^2^ Department of Pharmacy, Pengzhou Second People's Hospital Pengzhou China; ^3^ Department of Pharmacy, Pengzhou People's Hospital Pengzhou China; ^4^ Department of Pharmacy, Qionglai Maternal & Child Health and Family Planning Service Center Qionglai China; ^5^ Department of Pharmacy, Union Hospital, Tongji Medical College Huazhong University of Science and Technology Wuhan China

**Keywords:** adverse event, disproportionality analysis, FAERS database, hematological toxicities, PARP inhibitor

## Abstract

**Objective:**

Poly ADP‐ribose polymerase inhibitors (PARPis) have significantly improved clinical effects in gynecological oncology. However, PARPis could also induce severe organ system toxicities, including the hematological system. Our study aimed to extensively characterize the hematological toxicities of PARPis based on the real‐world data.

**Methods:**

Disproportionality analysis was used to evaluate the association between PARPis and hematotoxicity adverse events. Data were extracted from the US FDA Adverse Event Reporting System (FAERS) database between January 2015 and September 2021. The characteristics of PARPi‐associated hematological toxicities, and the onset time and fatality proportion were further analyzed.

**Results:**

Out of 24,045 adverse events reports, 4088 hematotoxicity reports (17.00%) were analyzed, with a median age of 64.95 (interquartile range [IQR] 51–71) years. All PARPis were detected with positive safety signals of hematological toxicities in four detection methods. Unexpected significant adverse events such as lymphadenopathy, lymphoedema, and metastases to lymph nodes might also occur. The median time‐to‐onset was 28 (IQR 10–101) days and the fatality proportion of hematological toxicities with PARPis was 8.76%, with a statistical difference in different PARPis.

**Conclusion:**

Hematological toxicities caused by PARPis preferred to occur early and might result in serious outcomes. Early identification and response to the PARPi‐related hematological toxicities were important and further basic research were needed to confirm the mechanism of results in this study.

## Graphical abstract

Our study extensively characterize the hematological toxicities of PARPis based on the FAERS real‐world data by disproportionality analyses. 4088 hematotoxicity reports (17.00%) were analyzed and all PARPis were detected with positive safety signals of hematological toxicities in four detection methods. Unexpected significant adverse events such as lymphadenopathy, lymphoedema, and metastases to lymph nodes might also occur. The median time‐to‐onset was 28 (IQR 10‐101) days and the fatality proportion of hematological toxicities with PARPis was 8.76%, with a statistical difference in different PARPis.

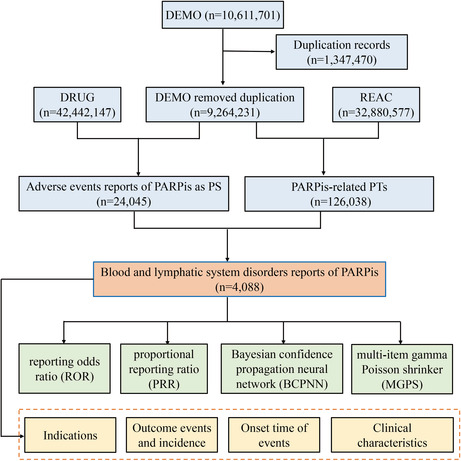

## 1 INTRODUCTION

Poly ADP‐ribose polymerase inhibitor (PARPi) is a potentially synthetic lethal effect agent for the therapy of cancers characterized by specific DNA‐repair defects, such as tumor cells that contain BRCA1 and/or BRCA2 (BRCA1/2) mutations and present defects in homologous recombination repair.^1^, ^2^, ^3^ In 2005, Bryant et al.^4^ and Farmer et al.^5^ confirmed for the first time that PARPis could produce joint lethal effect on tumors with BRCA mutation, which provided new landscape for tumor treatment. Due to the advantage of significant progression‐free survival (PFS) and relatively low incidence of serious side effects, PARPis are an ideal treatment option for both primary and relapsed ovarian cancer, and other cancers.^6^, ^7^, ^8^, ^9^ Olaparib, the world's first PARPi, was approved by the US Food and Drug Administration (FDA) for the treatment of ovarian cancer since 2014.^10^ Subsequently, the other PARPis to date rucaparib, niraparib, and talazoparib successively entered into clinical use in 2016, 2017, and 2018, respectively. PARPis have been continuously updated in the guidelines of major oncology associations and currently approved for treating ovarian cancer, breast cancer, pancreatic cancer, and prostate cancer in clinic.^8^, ^9^, ^11^, ^12^

Generally, the reporting peak of adverse drug reactions (ADRs) occurs 2 or 3 years after medication approval.^13^ The majority of current ADRs on PARPis are from case and clinical cohorts or case–control studies, and understanding of large‐sample ADRs in real‐world after marketing are far from sufficient and systematic.^14^, ^15^ In recent years, the potential adverse events (AEs) induced by PARPis have attracted extensive attention from scholars, including hematotoxicity, interstitial pneumonia, elevated serum creatinine and folic acid deficiency.^6^, ^15^, ^16^, ^17^ The incidence of hematological AEs continued to increase with the widespread use of PARPis. A systematic review and meta‐analysis of investigating all‐grade and G3‐G4 hematological AEs of maintenance therapy with olaparib indicated that the overall incidences of all‐grade and G3‐G4 AEs in olaparib group were 97.6% and 41%, respectively. Patients with olaparib treatment showed higher risk of all‐grade (RR = 3.39; 95%CI = 2.05–5.61, *p* < 0.00001) and G3‐G4 anemia (RR = 8.86; 95%CI = 4.12–19.07, *p* < 0.00001), all‐grade neutropenia (RR = 2.36; 95%CI = 1.49–3.74, *p* = 0.0003) and thrombocytopenia (RR = 3.52; 95%CI = 1.71–7.27, *p* = 0.0006).^18^ Several other meta‐analyses have reported the similar results.^14^, ^19^, ^20^ Additionally, the US Food and Drug Administration (FDA) has warned that PARPis might cause anemia, neutropenia, leukopenia, and thrombocytopenia in the label. Moreover, whether there are differences in hematotoxicity among different PARPis is not known. Therefore, the long‐term large‐sample post‐marketing risk studies of PARPis for hematological toxicities are urgently needed.

The spontaneous reporting System (SRS) covers tens of millions of case reports of adverse drug events (ADE), providing a huge source of data for real‐world safety issues and early risk identification related to drug therapy.^21^ The aim of our study was to assess PARPis‐induced hematotoxicity by a disproportionality analysis using the US FDA Adverse Event Reporting System (FAERS), which is a public database designed to conduct post‐marketing drug safety surveillance research.

## 2 METHODS

### 2.1 Data source

We conducted a retrospective pharmacovigilance study based on the FAERS database which was downloaded from the FDA website.^22^ FAERS is a reporting database voluntarily submitted by consumers, health professionals, pharmaceutical manufacturers and patients from different regions, and is updated quarterly, which is used to support the FDA's post‐marketing monitoring program for drugs and therapeutic biological products.^23^ The FAERS data documents included: DEMO (demographic and administrative information), DRUG (drug information), REAC (coded for the adverse events), OUTC (patient outcomes), RPSR (report sources), THER (therapy start dates and end dates for reported drugs), INDI (indications for drug administration) and deleted cases. In our study, data of PARPis were extracted during the period between January 2015 and September 2021 in the FAERS database, and all data were imported into MySQL 8.0 for further analysis. Necessary variables such as primaryid, caseid, drug_seq were also extracted from the database.

**FIGURE 1:**
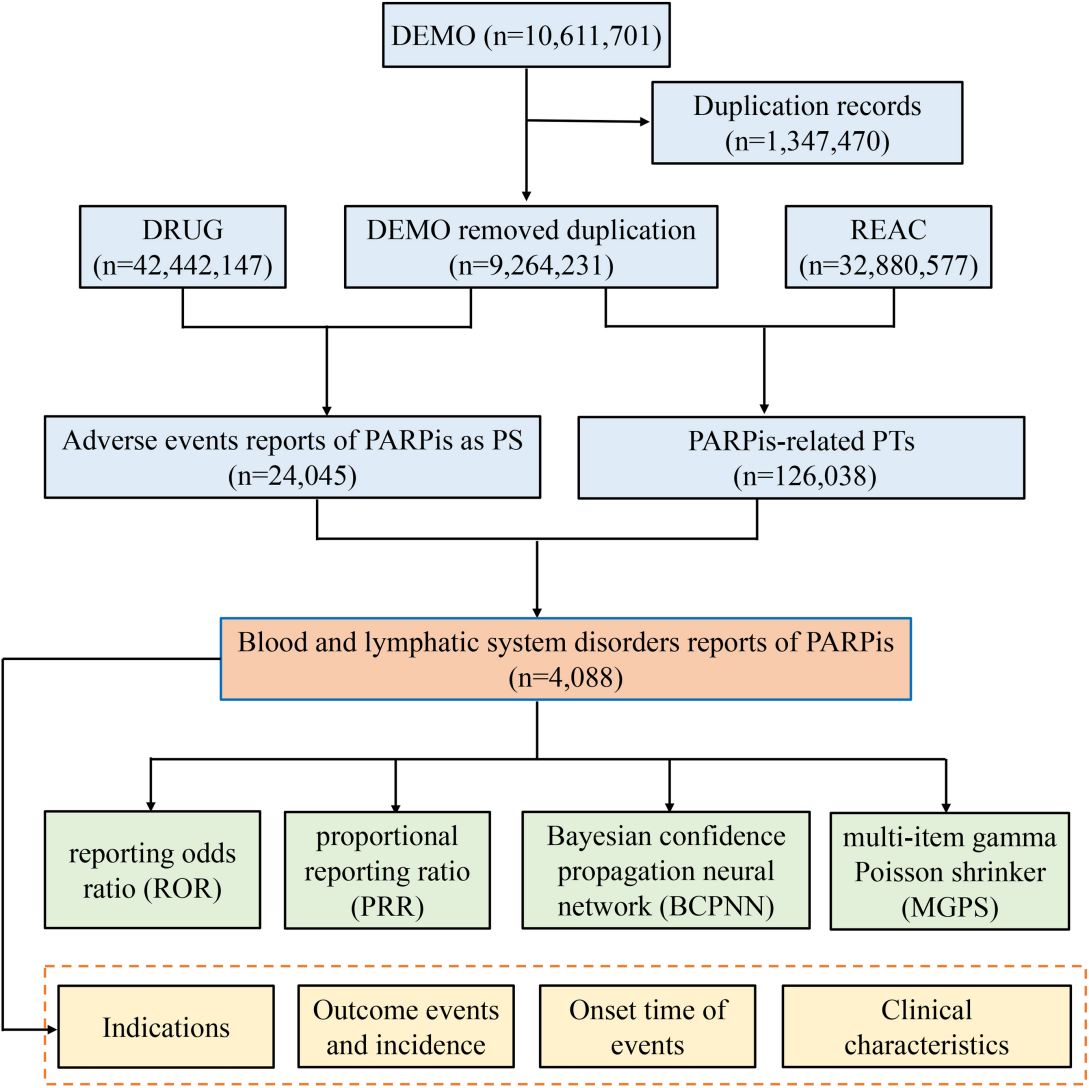
The flow diagram of selecting PARPis‐related AEs from FAERS database

In total, 10,611,701 reports were retrieved from the FAERS database. We used a two‐step deduplication process to ensure unique report. Expurgated data from the deleted file was downloaded from FAERS, and then performed the deduplication step following the FDA's recommendations, selecting the higher PRIMARYID when the CASEID and FDA_DT were the same and selecting the latest FDA_DT when the CASEIDs were the same,^24^ finally reducing the number of reports to 9,264,231 (Figure 1).

### 2.2 Data extraction

The generic and brand names of PARPis approved by FDA were used to identify AEs of PARPis in the DRUG files, including Olaparib (LYNPARZA), Niraparib (ZEJULA), Rucaparib (RUBRACA) and Talazoparib (TALZENNA) (Table S1). AEs in FAERS are coded by the preferred term (PT) from standardized Medical Dictionary for Regulatory Activities (MedDRA) terminology, which consists of 27 system organ classes (SOCs).^25^ The structural hierarchy of the MedDRA terminology has five levels: SOC (system organ class), HLGT (high level group term), HLT (high level term), PT (preferred term), and LLT (lowest level term).^26^ Further, a PT may be corresponding to multiple SOCs in MedDRA. Accordingly, the latest versions MedDRA 24.0 were used to classify AEs in reports to the relevant SOC level in MySQL 8.0. We analyzed all PARPis‐induced PTs in the FAERS database below the SOC of blood and lymphatic system disorders (SOC: 10005329). The role code about AEs had been assigned by reporters, including primary suspected (PS), secondary suspect drug (SS), concomitant (C), and interacting (I). To guarantee PARPis were the most likely to cause AEs during drug use, reports were reserved when the drug was considered as PS in DRUG files.^25^

Clinical characteristics (gender, age, reporting area, reporter and indication, etc.) of reports with PARPi‐associated hematological toxicities were analyzed, if they were available. Furthermore, we calculated the time‐to‐onset of hematological toxicities and the proportion of serious outcome of AEs that were caused by different PARPis. The calculation method of the onset time is the interval between START_DT (start time of PARPis use) and EVENT_DT (time of AE occurrence). Reports with date errors (START_DT later than EVENT_DT), inaccurate time entries and missing specific data were excluded. Severe outcomes mainly included life‐threatening events or those causing hospitalization, disability, or death. The calculation of the proportion was dividing the number of serious outcomes reports by the total reports.^27^

### 2.3 Data mining

Disproportionality analyses were performed to detect potential signals of hematological AEs caused by total/specific PARPis.^23^ It compares the proportion of hematological toxicities reports among the PARPis with the proportion of hematological toxicities reports in all other drugs.^23^ The general principle is that a signal is considered to have been generated in the data extraction period, when the specific AE occurrence rate of a specific drug is significantly stronger than most other drugs in the database and reaches a certain threshold or criteria. Both Frequentist and Bayesian methods in the disproportionality analysis were applied to investigate the correlation between an AE and the drug, using the reporting odds ratio (ROR),^28^ the proportional reporting ratio (PRR),^29^ the information component (IC)^30^ and the empirical bayes geometric mean (EBGM).^31^ Each algorithm has its own advantages and no one algorithm is universally better than others. To improve the accuracy of signals and eliminate some false positive PTs, the AEs signals could be detected when they met the four algorithm criteria simultaneously in our study. The equations and corresponding thresholds of the four algorithms are listed in Table S2.

Descriptive analysis was employed to summarize the clinical characteristics of PARPis‐associated hematological toxicities in the FAERS database. Kruskal‐Wallis test was used to compare the time‐to‐onset of hematological toxicities in different PARPi regimens. The fatality proportion of hematological toxicities in different PARPi regimens was compared using Fisher's exact test or Pearson's chi‐squared test. The *p* < 0.05 between PARPis regimens indicated a statistical difference. All data extraction and statistical analyses were performed by MYSQL 8.0, SPSS 24.0, Microsoft EXCEL 2019 and the GraphPad Prism 8 (GraphPad Software).

## 3 RESULTS

### 3.1 Descriptive analysis

From January 2015 to September 2021, a total of 24,045 PARPis‐associated AEs reports were recorded, in which 4088 reports of hematological toxicities were identified. The clinical characteristics of reports were summarized in Table 1. The number of hematological toxicities reports had significantly increased during the study period, reflecting the increasingly post‐marketing clinical application of PARPis. Females (82.36%, valid reports in 3522/4088) were more likely to report hematological toxicities. The median age of reports was 64.95 years (interquartile range [IQR] 56–71, valid reports in 2267/4088). The reports were mainly from America (64.75%), followed by Japan (9.83%), and France (5.28%). And 48.14% (valid reports in 3673/4088) of the reports were submitted by healthcare professionals. The median time‐to‐onset of reports was 28 (IQR 10–101, valid reports in 1807/4088) days and 51.69% of the hematological toxicities occurred within 30 days after excluded invalid reports. Hematological toxicities AEs were most frequently reported in ovarian cancer patients (67.98%). The most serious outcome event was hospitalization (28.82%), and the death in all cases was 8.76%. The majority of hematological toxicities cases were reported with niraparib (44.55%), followed by olaparib (33.02%), and rucaparib (18.74%).

**TABLE 1:** Clinical characteristics of reports with PARPi‐associated hematological toxicities in the FAERS database (January 2015 to September 2021)

Characteristics	Report number, *n*	Report proportion, %
Number of reports	4088	
Gender		
Female	3367	82.36
Male	155	3.79
Unknown or missing	566	13.85
Age (years)		
<18	6	0.15
18 ≤ and ≤ 65	1195	29.23
>65	1066	26.08
Unknown or missing	1821	44.55
Median (IQR)	64.95 (51–71)	/
PARPis as suspected drug		
Olaparib	1350	33.02
Niraparib	1821	44.55
Rucaparib	766	18.74
Talazoparib	151	3.69
The time to onset (days)		
0–30	934	22.85
31–60	206	5.04
61–90	176	4.31
91–180	175	4.28
181–360	128	3.13
>361	188	4.60
Unknown or missing	2281	55.80
Median (IQR)	28 (10–101)	/
Indications		
Ovarian cancer	2779	67.98
Breast cancer	189	4.62
Others	786	19.23
Unknown or missing	334	8.17
Serious outcome		
Death	358	8.76
Life‐threatening	414	10.13
Hospitalization	1178	28.82
Disability	35	0.86
Others	2751	67.29
Reported Countries (Top five)		
America	2647	64.75
Japan	402	9.83
France	216	5.28
Italy	103	2.52
Germany	92	2.25
Reported Person		
Healthcare profession		
Physician	1175	28.74
Pharmacist	111	2.72
Other health‐professional	723	17.69
Non‐healthcare professional		
Consumer	1664	40.70
Unknown	415	10.15
Reporting year		
2021 Q3^a^	871	21.31
2020	1013	24.78
2019	836	20.45
2018	836	20.45
2017	364	8.90
2016	111	2.72
2015	57	1.39

^a^ The third quarter of 2021. IQR, interquartile range.

### 3.2 Signal values associated with different PARPis

Within the SOC level (Table 2), blood and lymphatic system disorders in all PARPis were overreported compared to the background frequency (ROR 4.09, PRR 3.56, IC 1.82, EBGM 3.54). The majority of PARPis‐associated blood and lymphatic system disorders were reported by niraparib (44.55%, ROR 3.92, PRR 3.44, IC 1.78, EBGM 3.43), followed by olaparib (33.02%, ROR 5.32, PRR 4.40, IC 2.14, EBGM 4.39). Every PARPi presented significant positive signals in blood and lymphatic system disorders in four algorithm criteria. Moreover, although talazoparib contributed the lowest percentage of reports, it exhibited the strongest association with hematological toxicities AEs, owing to its highest ROR (9.81), PRR (6.89), IC (2.78), and EBGM (6.89).

**TABLE 2:** Signal detection for PARPi‐associated hematological toxicities

PARPI	*N*	ROR (95% CI)	PRR (χ2)	IC (IC025)	EBGM (EBGM05)
All PARPIs	4088	4.09 (3.95–4.23)	3.56 (7845.89)	1.82 (1.77)	3.54 (3.42)
Olaparib	1350	5.32 (5.01–5.64)	4.40 (3720.16)	2.14 (2.04)	4.39 (4.14)
Niraparib	1821	3.92 (3.73–4.13)	3.44 (3300.2)	1.78 (1.70)	3.43 (3.26)
Rucaparib	766	2.82 (2.61–3.04)	2.59 (785.04)	1.37 (1.26)	2.59 (2.40)
Talazoparib	151	9.81 (8.07–11.92)	6.89 (798.75)	2.78 (2.45)	6.89 (5.67)

We also detected a class of specific hematological AEs spectrum in different PARPi strategies, and the positive signals were showed in Table 3. Anemia (1831, 44.79%) is the hematological AEs that most frequently reported in PARPis. All hematological toxicities in the label of PARPis were found in our study. However, neutropenia (299, 7.31%) and febrile neutropenia (110, 2.69%) were reported in FAERS, which were also listed in the drug label, but the threshold of at least one of the four methods cannot be met. Finally, 20 disproportionality signals were detected for both olaparib and niraparib, and 7, 9 for rucaparib and talazoparib, respectively. Of note, a lot of significant new AEs that unrecorded in PARPis label were found in our data mining, such as metastases to lymph nodes (29, 2.15%), lymphangiosis carcinomatosa (7, 0.52%), macrocytosis (5, 0.37%) for olaparib; lymphadenopathy (124, 6.81%), petechiae (85, 4.67%), lymphoedema (30, 1.65%), increased tendency to bruise (26, 1.43%), metastases to lymph nodes (24, 1.32%), transfusion reaction (9, 0.49%), abdominal lymphadenopathy (8, 0.44%), metastases to spleen (6, 0.33%) for niraparib; lymphadenopathy (31, 4.05%), lymphoedema (8, 1.04%) for rucaparib.

**TABLE 3:** Signal strength of hematological toxicities reports of PARPis at the Preferred Term (PT) level in FAERS database

PARPIs	Preferred term (PT)	The report number	ROR (95% CI)	PRR (χ2)	IC (IC025)	EBGM (EBGM05)
Olaparib	Anemia	606	12.23 (11.25–13.31)	11.17 (5616.08)	3.47 (3.32)	11.09 (10.20)
	Myelodysplastic syndrome	184	49.51 (42.66–57.46)	48.11 (8220.87)	5.54 (5.00)	46.60 (40.15)
	Thrombocytopenia	128	4.26 (3.58–5.08)	4.20 (312.19)	2.07 (1.76)	4.19 (3.51)
	Acute myeloid leukemia	113	27.57 (22.85–33.26)	27.10 (2789.80)	4.73 (4.15)	26.62 (22.06)
	Pancytopenia	111	7.69 (6.38–9.29)	7.58 (632.02)	2.92 (2.54)	7.54 (6.25)
	Bone marrow failure	71	8.92 (7.05–11.28)	8.83 (490.55)	3.13 (2.62)	8.78 (6.95)
	Hematotoxicity	47	19.00 (14.23–25.36)	18.86 (785.19)	4.22 (3.31)	18.63 (13.96)
	Myelosuppression	46	13.71 (10.25–18.35)	13.62 (533.28)	3.76 (2.96)	13.50 (10.09)
	Metastases to lymph nodes^a^	29	15.97 (11.07–23.04)	15.90 (400.68)	3.98 (2.81)	15.74 (10.91)
	Leukemia	18	6.16 (3.88–9.80)	6.15 (77.32)	2.62 (1.51)	6.13 (3.85)
	Cytopenia	14	4.07 (2.41–6.88)	4.06 (32.27)	2.02 (0.88)	4.06 (2.40)
	Hemolytic anemia	12	4.97 (2.82–8.77)	4.96 (37.87)	2.31 (0.97)	4.95 (2.81)
	Acute leukemia	12	29.76 (16.79–52.73)	29.71 (326.19)	4.86 (2.25)	29.13 (16.44)
	Blood disorder	10	4.47 (2.40–8.33)	4.47 (26.86)	2.16 (0.71)	4.46 (2.40)
	Anemia macrocytic	8	16.27 (8.10–32.67)	16.25 (113.22)	4.01 (1.39)	16.08 (8.01)
	Lymphangiosis carcinomatosa^a^	7	22.11 (10.48–46.65)	22.08 (138.80)	4.44 (1.31)	21.77 (10.31)
	Bone marrow disorder	6	7.83 (3.51–17.48)	7.83 (35.54)	2.96 (0.58)	7.79 (3.49)
	Macrocytosis^a^	5	15.65 (6.48–37.79)	15.64 (67.78)	3.95 (0.62)	15.48 (6.41)
	Aplasia pure red cell	5	6.78 (2.82–16.34)	6.78 (24.52)	2.76 (0.23)	6.75 (2.80)
	Myeloid leukemia	4	32.38 (12.02–87.25)	32.36 (118.91)	4.99 (0.37)	31.67 (11.76)
Niraparib	Anemia	726	8.23 (7.63–8.88)	7.76 (4269.84)	2.94 (2.82)	7.69 (7.13)

Thrombocytopenia	573	11.53 (10.59–12.55)	10.98 (5155.95)	3.44 (3.29)	10.85 (9.97)

Lymphadenopathy^a^	124	7.50 (6.28–8.96)	7.42 (684.21)	2.88 (2.54)	7.37 (6.17)

Pancytopenia	118	4.70 (3.92–5.64)	4.66 (338.56)	2.22 (1.89)	4.64 (3.87)

Petechiae^a^	85	16.92 (13.64–20.98)	16.79 (1238.25)	4.04 (3.47)	16.48 (13.29)

Bone marrow failure	68	4.92 (3.87–6.24)	4.89 (209.64)	2.28 (1.83)	4.87 (3.83)

Myelodysplastic syndrome	45	6.67 (4.97–8.94)	6.64 (214.15)	2.72 (2.09)	6.60 (4.92)

Acute myeloid leukemia	31	4.25 (2.99–6.06)	4.24 (76.55)	2.08 (1.38)	4.23 (2.97)

Lymphoedema^a^	30	8.76 (6.11–12.56)	8.74 (203.63)	3.11 (2.22)	8.66 (6.04)

Platelet disorder	28	21.88 (15.03–31.86)	21.83 (542.43)	4.41 (3.04)	21.30 (14.63)

Increased tendency to bruise^a^	26	5.10 (3.46–7.50)	5.09 (84.91)	2.34 (1.52)	5.06 (3.44)

Metastases to lymph nodes^a^	24	7.62 (5.09–11.39)	7.60 (136.40)	2.91 (1.93)	7.54 (5.04)

Bone marrow disorder	20	15.32 (9.84–23.85)	15.29 (262.42)	3.91 (2.45)	15.04 (9.66)

Blood disorder	20	5.20 (3.35–8.07)	5.19 (67.25)	2.37 (1.39)	5.16 (3.33)

Myelosuppression	17	3.42 (2.12–5.51)	3.42 (28.97)	1.77 (0.80)	3.41 (2.12)

White blood cell disorder	11	7.33 (4.05–13.28)	7.33 (59.60)	2.86 (1.26)	7.27 (4.02)

Transfusion reaction^a^	9	26.86 (13.83–52.18)	26.84 (216.97)	4.70 (1.76)	26.04 (13.41)

Red blood cell abnormality	8	16.65 (8.27–33.53)	16.64 (115.29)	4.03 (1.39)	16.33 (8.11)

Abdominal lymphadenopathy^a^	8	15.11 (7.51–30.41)	15.10 (103.47)	3.89 (1.35)	14.85 (7.38)

Metastases to spleen^a^	6	26.48 (11.75–59.7)	26.47 (142.52)	4.68 (1.09)	25.69 (11.39)
Rucaparib	Anemia	438	8.93 (8.10–9.84)	8.36 (2848.80)	3.06 (2.89)	8.32 (7.55)
	Thrombocytopenia	146	5.07 (4.30–5.97)	4.97 (463.93)	2.31 (2.02)	4.96 (4.21)
	Bone marrow failure	36	4.66 (3.35–6.46)	4.63 (102.43)	2.21 (1.55)	4.62 (3.33)
	Lymphadenopathy^a^	31	3.32 (2.33–4.73)	3.31 (49.88)	1.72 (1.06)	3.30 (2.32)
	Blood disorder	10	4.65 (2.50–8.65)	4.64 (28.49)	2.21 (0.75)	4.63 (2.49)
	Lymphoedema^a^	8	4.16 (2.07–8.32)	4.15 (19.09)	2.05 (0.43)	4.14 (2.07)
	Bone marrow disorder	8	10.87 (5.42–21.80)	10.86 (71.09)	3.43 (1.18)	10.79 (5.38)
Talazoparib	Anemia	61	17.93 (13.69–23.48)	15.66 (843.98)	3.97 (3.25)	15.65 (11.95)
	Thrombocytopenia	36	17.85 (12.70–25.09)	16.52 (527.04)	4.05 (3.00)	16.51 (11.74)
	Pancytopenia	25	25.17 (16.82–37.68)	23.85 (547.89)	4.57 (3.02)	23.82 (15.91)
	Neutropenia	19	7.53 (4.76–11.92)	7.26 (103.05)	2.86 (1.72)	7.25 (4.58)
	Febrile neutropenia	16	12.76 (7.74–21.01)	12.34 (167.16)	3.62 (2.07)	12.34 (7.49)
	Leukopenia	8	8.11 (4.03–16.32)	7.98 (48.96)	3.00 (0.97)	7.98 (3.97)
	Hematotoxicity	8	45.34 (22.52–91.29)	44.56 (340.07)	5.47 (1.73)	44.47 (22.08)
	Myelodysplastic syndrome	6	21.60 (9.65–48.35)	21.32 (116.18)	4.41 (1.04)	21.30 (9.52)
	Cytopenia	4	16.39 (6.12–43.89)	16.26 (57.27)	4.02 (0.23)	16.25 (6.07)

Abbreviations: CI, confidence interval; EBGM, empirical Bayesian geometric mean; IC, information component; PRR, proportional reporting ratio; ROR, reporting odds ratio; χ^2^, chi‐squared.

^a^ Emerging findings of PARPis‐related AEs in FAERS database.

### 3.3 Time‐to‐onset and fatality proportion

**FIGURE 2:**
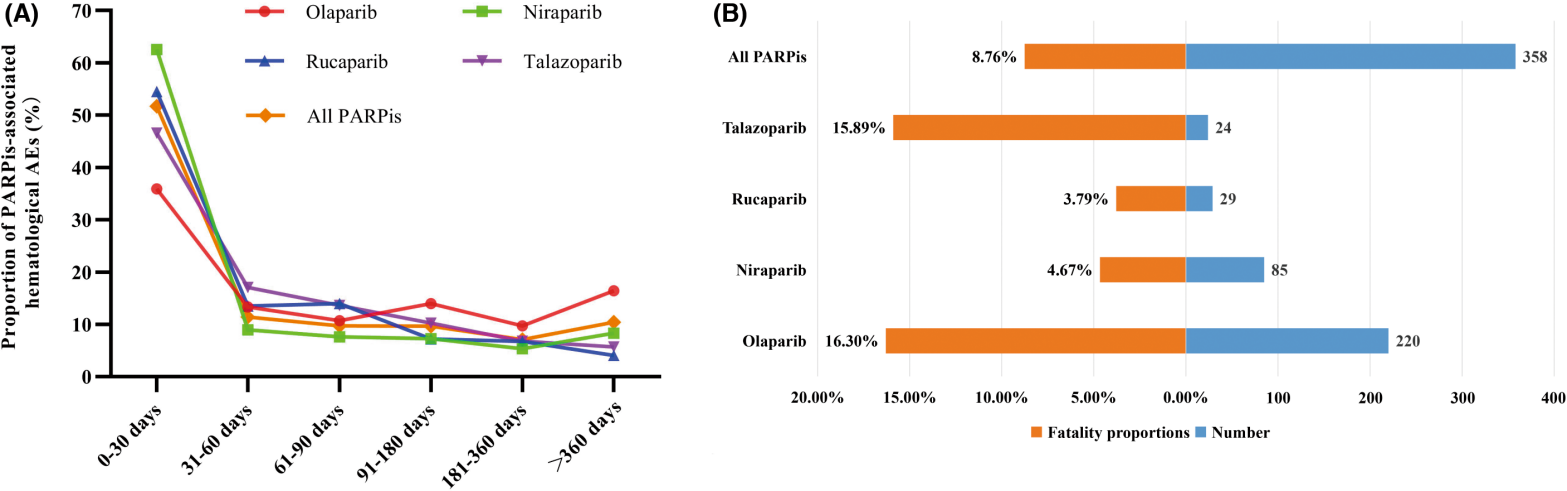
(A) Time to onset of PARPis‐related hematological AEs. (B) The case fatality rate for PARPis‐related hematological AEs

The onset time of blood and lymphatic system disorders for each PARPi regimen was showed in Figure 2A. The study found the onset time of PARPis had statistical difference (*p* < 0.001). The longest median onset time was 63 (IQR 16–189) days for olaparib, while the shortest of 21 (IQR 9–74) days for niraparib, and 22.5 (IQR 2–75) days for rucaparib, 35.5 (IQR 14–84.5) days for talazoparib, respectively.

To explore the prognosis of reports with hematological AEs after the use of PARPis, our study evaluated the outcome of reports by fatality proportions. The corresponding results were shown in Table 1 and Figure 2B, and we found a statistical difference in fatality proportions of PARPi‐associated hematological AEs among PARPi regimens (*p* < 0.001). In all PARPis, olaparib (16.30%, 220 deaths out of 1350 cases) had the highest fatality proportion of PARPi‐associated hematological AEs, followed by talazoparib (15.89%, 24 deaths out of 151 cases), niraparib (4.67%, 85 deaths out of 1821 cases), and the lowest for rucaparib (3.79%, 29 deaths out of 766 cases).

## 4 DISCUSSION

In addition to the significant survival benefits in clinical practice, PARPis can also substantially increase the risk of organ system toxicities such as hematological toxicities. However, most RCTs (randomized controlled trials) of PARPis have identified clinical benefits rather than AEs and just a brief description of the severe or even fatal AEs were given.^10^, ^32^, ^33^, ^34^, ^35^, ^36^ To the best of our knowledge, this is the first large‐sample real‐world pharmacovigilance study on hematological AEs following the use of post‐marketing PARPis based on the FAERS database. After deduplication and data analysis, we detected that the clinical application of PARPis were confirmed positive for association with adverse events in blood and lymphatic system disorders. In the meanwhile, the characterization of PARPis‐related hematological toxicities reports were described in detail. The significant hematological‐associated AE signals with different PARPis were identified based on our analysis of the FAERS database. Our results could help physicians and patients to well understand the hemotoxicity spectrums for different PARPis and reduce potential medication risks.

And 4088 cases of PARPi‐associated blood and lymphatic system disorders were included, with the largest collection of cases to date. The increased reports year by year may be mainly because of the widespread clinical application of PARPis and the increased awareness of post‐marketing drug safety supervision by healthcare professionals. PARPi‐associated AEs predominately affected female (95.60%, 3367/3522), which were consistent with the indication of ovarian cancer (74.03%, 2779/3754). Although most PARPi‐associated cases collected from the United States, hematological AEs showed the low composition ratio (13.56%, 2647/19522). The race relations with the incidence of hematological AEs caused by PARPis was still not clear. However, our study suggested that the Asia (36.41%, 402/1104) and Europe (36.63%, 409/1122) should pay more attention to the risk of hematological toxicities caused by PARPis because of the high hematological AEs proportion in FAERS. It is vital to monitor hematological toxicities in clinical practice, and provide guidance to the use of PARPis. If hematologic toxicity persists, dose adjustment may be required and a full blood count should be monitored weekly until the toxicity is resolved. Once diagnosed with myelodysplastic syndrome (MDS) or acute myeloid leukemia (AML), PARPi regimens should be discontinued and appropriate treatment should be taken.^11^

The most frequently reported PARPi‐induced hematological AE was anemia in the real world and anemia, thrombocytopenia, myelosuppression were the same disproportionality signals in all PARPis. Studies have showed that PARP1 regulates cell differentiation in the bone marrow or blood system^37^ and PARP2 plays a role in regulating erythropoiesis.^38^ It is suggested that PARPis may lead to hematological toxicities by inhibiting the expression of PARP1 and PARP2. As these studies are based on animals in vivo, the hematological mechanism of PARP enzyme system in human remains unclear, so relevant studies need to be further clarified. Notably, hematological AEs had been reported in many case reports and RCTs as the most common AEs of grade 3 or worse, and anemia was the most common hematological toxicity.^10^, ^32^, ^33^, ^34^, ^35^, ^36^ Ruiz‐Schutz et al. in 2019 investigated the risk of hematologic toxicities on olaparib and demonstrated that olaparib treatment was associated with a significant increase in the risk of developing all‐grade and high‐grade anemia.^14^ Furthermore, a meta‐analysis also revealed a distinct increased risk of severe anemia (RR = 2.21, 95% CI = 1.53–3.49, *p* < 0.001) in olaparib therapy.^20^ These results were consistent with ours. The number of significant signals in rucaparib (*n* = 7) and talazoparib (*n* = 9) seemed to be less than olaparib (*n* = 20) and niraparib (*n* = 20). Interestingly, although talazoparib was the last PARPi approved by FDA for treating metastatic breast cancer in 2018 and limited toxicity‐associated reports were accessible, it showed overreporting in hematological toxicities according to disproportionality analysis. Unexpected significant AEs that uncovered in the drug label were mainly focused on the lymphatic system in our data mining, such as lymphadenopathy and lymphoedema, and toxicity of lymphatic might be considered as a potentially existing toxicity for PARPis. Importantly, with the widespread use of PARPis in cancer patients, it is necessary for clinicians to master the increasing hematological toxicities that occured in PARPis.

Our studies suggested PARPi‐associated hematological toxicities occurred 30 days (934, 51.69%) after the initiation of PARPis, and the median time‐to‐onset was 28 (IQR 10–101) days with significant difference between different PARPi regimens. Olaparib had the longest median onset time in PARPis, and 40.09% of hematological AEs were still reported after 90 days of medication. A systematic review consistent with our results,^39^ showed hematological toxicities tended to occur early after treatment initiation with recovery after a few months, which were the most common cause of dose modification, interruption, and discontinuation. Thus, clinicians should pay more attention to the PARPis with a short onset time of hematological toxicities, and further studies are necessary to explain this phenomenon. A case may have multiple outcomes in FAERS, such as the death may experience the disability and life‐threatening at the same time. The risk of resulting in death of PARPi‐associated hematological toxicities was explored, and the outcome of 358 cases (8.76%) was death. It was noteworthy that talazoparib had the high mortality (15.89%, 24/151), in spite of the lowest reports in PARPis. We observed that the fatality rates had statistical significance in different PARPis. SOLO2 trial showed that AEs with an outcome of death occurred in five (3%) of 196 patients in the olaparib group after the safety follow‐up period, which were all attributed to MDS or AML.^11^, ^40^ According to our data, almost all patients used PARPis for treating cancers, so death may be also attributed to tumor progression rather than only the toxicity of PARPis. However, even if the death was caused by disease progression or other causes, the case also experienced hematotoxicity in our study. Our results revealed that the death of AEs were statistically significantly associated with PARPi, consistent with SOLO2 trial showing all deaths in the olaparib group were caused by hematotoxicity (myelodysplastic syndrome and acute myeloid leukemia). Unfortunately, due to the limitations of FAERS, we could not determine through data mining whether the hematotoxicity‐related death was caused by PARPi or disease progression. Therefore, the causality should be confirmed by further experimental exploration, clinical trials, case–control or cohort studies. Besides, it seems more likely that the outcome of hematological toxicities is termination of PARPis treatment, accelerating disease progression. The proportion of treatment termination due to severe hematotoxicity was 35% (olaparib)^41^ and 10% (niraparib),^33^ respectively.

Although our study had significant advantages based on FAERS database and the data mining technology, this research existed a few limitations shared by all pharmacovigilance databases. First, FAERS database is a self‐reporting system with reporting randomness (e.g., existing selective, incomplete, inaccurate, untimely, and unverified reporting) and a massive loss of data (e.g., gender and age). It is extremely difficult to consider confounders simultaneously such as dosage, duration of use, comorbidities, drug combinations, and other factors that may influence the occurrence of hematotoxicity. Second, the FAERS database contains only cases with adverse events. The incidence rate of each AEs cannot be calculated because of lacking total numbers of patients receiving PARPis treatment, i.e., lacking the denominator of drug exposure. Third, we focus only on hematological toxicity, and the deep relationship between PARPi and other system organ classes remains unknown. Further clinical investigation and experimental studies are needed to confirm our results. Finally, disproportionality analysis based on FAERS neither existed causality nor quantified risk due to lacking pharmacological mechanism study, but only showing an evaluation of the signals strength, which was just a statistical association.^42^ Like other pharmacovigilance studies, Frequentist and Bayesian are indexes of increased risk in AE reports,^43^ whether a causality exists and also needs to be further validated by basic research.

## 5 CONCLUSION

Our pharmacovigilance analysis explored reports of hematological AEs caused by PARPis in the FAERS database. In total, 4088 hematotoxicity‐associated reports induced by PARPis were retrieved, and the signals were screened by disproportionality analysis. Common anemia, myelodysplastic syndrome, thrombocytopenia, and acute myeloid leukemia should be a cause for high concern. This study showed the PARPi‐associated hematological toxicities occurred early during the whole treatment and might develop into serious outcomes. The range of hematological toxicity, including the proportion of death that are not negligible in reported results, should be taken into account in clinical nursing and the design of RCTs. Further studies are needed to elucidate the underlying mechanisms of hematological toxicities caused by PARPis.

## AUTHOR CONTRIBUTIONS

Qilin Zhang and Yamin Shu contributed to conception and study design, and took responsibility for the collection, integrity, and accuracy of the data. All authors drafted the manuscript, participated in data analyses and interpretation, and revisions of the manuscript and approved the final version.

## FUNDING INFORMATION

This study was supported by grants from National Natural Science Foundation of China (No. 82104476).

## CONFLICTS OF INTEREST

The authors declare no conflicts of interest.

## ETHICS STATEMENT

No animal studies and human participants are presented in this manuscript.

## Supporting information


Table S1
Table S2

## Data Availability

The datasets generated during and/or analyzed during the current study are available from the corresponding author on reasonable request.
