# Supplementary material for: Hematological toxicities in PARP inhibitors: A real‐world study using FDA adverse event reporting system (FAERS) database
Source: Cancer Med. 2022 Jul 24;12(3):3365–75. doi: 10.1002/cam4.5062 (PMC9939145; doi:10.1002/cam4.5062)
Supplement: Supplementary file 1 — Table S1 Table S2 [file CAM4-12-3365-s001.docx]

**Supplementary Table 1** Summary of FDA-approved PARP inhibitors

| Target | Generic name | Brand name | Approval date |
| --- | --- | --- | --- |
| PARPis | Olaparib | LYNPARZA | 12/19/2014 |
|  | Niraparib | ZEJULA | 03/27/2017 |
|  | Rucaparib | RUBRACA | 12/19/2016 |
|  | Talazoparib | TALZENNA | 10/16/2018 |

**Supplementary Table 2.** Four major algorithms used for signal detection.

| Algorithms | Equation | Criteria |
| --- | --- | --- |
| ROR | ROR=ad/b/c | lower limit of 95% CI>1, N≥3 |
|  | 95%CI=e^ln(ROR)±1.96(1/a+1/b+1/c+1/d)^0.5^ |  |
| PRR | PRR=a(c+d)/c/(a+b) | PRR≥2, χ^2^≥4, N≥3 |
|  | χ^2^=[(ad-bc)^2](a+b+c+d)/[(a+b)(c+d)(a+c)(b+d)] |  |
| BCPNN | IC=log_2_a(a+b+c+d)(a+c)(a+b) | IC025>0 |
|  | 95%CI= E(IC) ± 2V(IC)^0.5 |  |
| MGPS | EBGM=a(a+b+c+d)/(a+c)/(a+b) | EBGM05>2 |
|  | 95%CI=e^ln(EBGM)±1.96(1/a+1/b+1/c+1/d)^0.5^ |  |

Equation: a, number of reports containing both the target drug and target adverse drug reaction; b, number of reports containing other adverse drug reaction of the target drug; c, number of reports containing the target adverse drug reaction of other drugs; d, number of reports containing other drugs and other adverse drug reactions. 95%CI, 95% confidence interval; N, the number of reports; χ^2^, chi-squared; IC, information component; IC025, the lower limit of 95% CI of the IC; E(IC), the IC expectations; V(IC), the variance of IC; EBGM, empirical Bayesian geometric mean; EBGM05, the lower limit of 95% CI of EBGM.
